# Comparative epigenomics indicate a common origin of ectopic and intrasellar corticotroph pituitary neuroendocrine tumors/adenomas: a case report

**DOI:** 10.1007/s00428-024-03760-5

**Published:** 2024-02-12

**Authors:** Alonso Barrantes-Freer, Max Braune, Benjamin Sandner, Matthias Dottermusch, Dirk Lindner

**Affiliations:** 1 Paul-Flechsig-Institute of Neuropathology, University Hospital Leipzig, Leipzig, Germany; 2 Medical Department III - Endocrinology, Nephrology, Rheumatology, University Hospital Leipzig, Leipzig, Germany; 3 Institute of Neuropathology, University Medical Center Hamburg-Eppendorf, Hamburg, Germany; 4 Department of Neurosurgery, University Hospital Leipzig, Leipzig, Germany

**Keywords:** Ectopic PitNET/adenoma, Gonadotroph PitNET/adenoma, 850 K, Genome-wide DNA methylation analysis

## Abstract

Ectopic pituitary neuroendocrine tumors (PitNET)/adenomas are rare and diagnostically challenging extra-sellar tumors. Previous studies have demonstrated the impact of epigenomic analyses in the diagnostics of sellar neoplasms and characterized the close relationship of epigenomic signatures and cellular origins of PitNET/adenomas. As of today, little is known about the pathogenesis of ectopic PitNET/adenomas, and epigenomic analyses have not been performed in these rare tumors. We report on the clinical course of an 81-year-old patient with sphenoid ectopic sparsely granulated corticotroph PitNET/adenoma and deploy genome-wide DNA methylation analysis to compare its methylation profile to a reference cohort of sellar neoplasms. Genome-wide methylation analysis revealed an epigenomic profile analogous to reference sellar corticotroph PitNET/adenomas, and the copy number variation profile showed loss of chromosomes 18 and 22. The methylation profile shows concordance with sellar corticotroph PitNET/adenomas suggesting a common cellular origin and confirming the reliability of methylation analyses as a diagnostic method in these rare tumors. This is the first data suggesting that epigenetic profiles of ectopic PitNET/adenoma do not differ from their sellar counterparts.

## Introduction

Ectopic pituitary neuroendocrine tumors (PitNET)/adenomas are defined as extrasellar well-differentiated pituitary neuroendocrine tumors that show no connection with intrasellar components [1, 2]. Ectopic PitNET/adenoma is a very rare entity with about 190 published cases [2]. The tumors are predominantly extracranial (~ 70%) [2, 3] and most commonly localize in the sphenoid sinus or sphenoid bone followed by the nasal cavity nasopharynx, clivus, and petrous temporal bone [1–12].

The origin of ectopic PitNET/adenoma is somewhat controversial [2, 13]. The common localization along the migration path of the Rathke’s pouch suggests an origin in embryologic remnants within the craniopharingeal duct [3, 14–16]. This view is further supported by the occasional finding of an empty sella especially in the context of sinus tumors [12, 17]. Also, the higher prevalence of ectopic PitNET in the sphenoid sinus and suprasellar region, corresponding to the start and end migration sites respectively, seems to suggest a migration defect, yet the exact mechanism is unknown [2]. Alternatively, an origin in the pharyngeal hypophysis has also been proposed [5, 14, 18].

Irrespective of their localization, ectopic PitNET/adenomas show morphological and immunohistochemical characteristics of types and subtpyes otherwise described in sellar PitNET/adenomas [5, 7, 16, 19]. Morphologically, they can show various and even mixed architectural patterns, thus posing a diagnostic challenge [13, 16]. Nevertheless, the majority of ectopic PitNET/adenomas express pituitary hormones, with PRL and ACTH being the most common in 15–40% [5, 12, 19] and frequently show a functional hormonal expression, more so than their sellar counterparts [19], which may be useful in the diagnostic workup of these lesions.

Although most ectopic tumors harbor a good prognosis [16], locally aggressive growth has been described [3, 13, 19]. In particular, ectopic tumors of the sphenoid sinus and clivus show a higher incidence of invasion of surrounding bone with extension to cavernous sinus or encasement of adjacent internal carotid arteries [2].

The ectopic localization, low frequency and variable morphology, and immunohistochemical profile often pose a diagnostic challenge for which more refined molecular analyses would be required [19]. Epigenomic analyses pose a powerful tool to classify tumors based on their cellular origins and have previously been applied in the field of pituitary tumors [20–24]. Epigenomic profiling of ectopic PitNET/adenomas might shed light on developmental characteristics and mechanisms of tumorigenesis.

## Case report

**Fig. 1:**
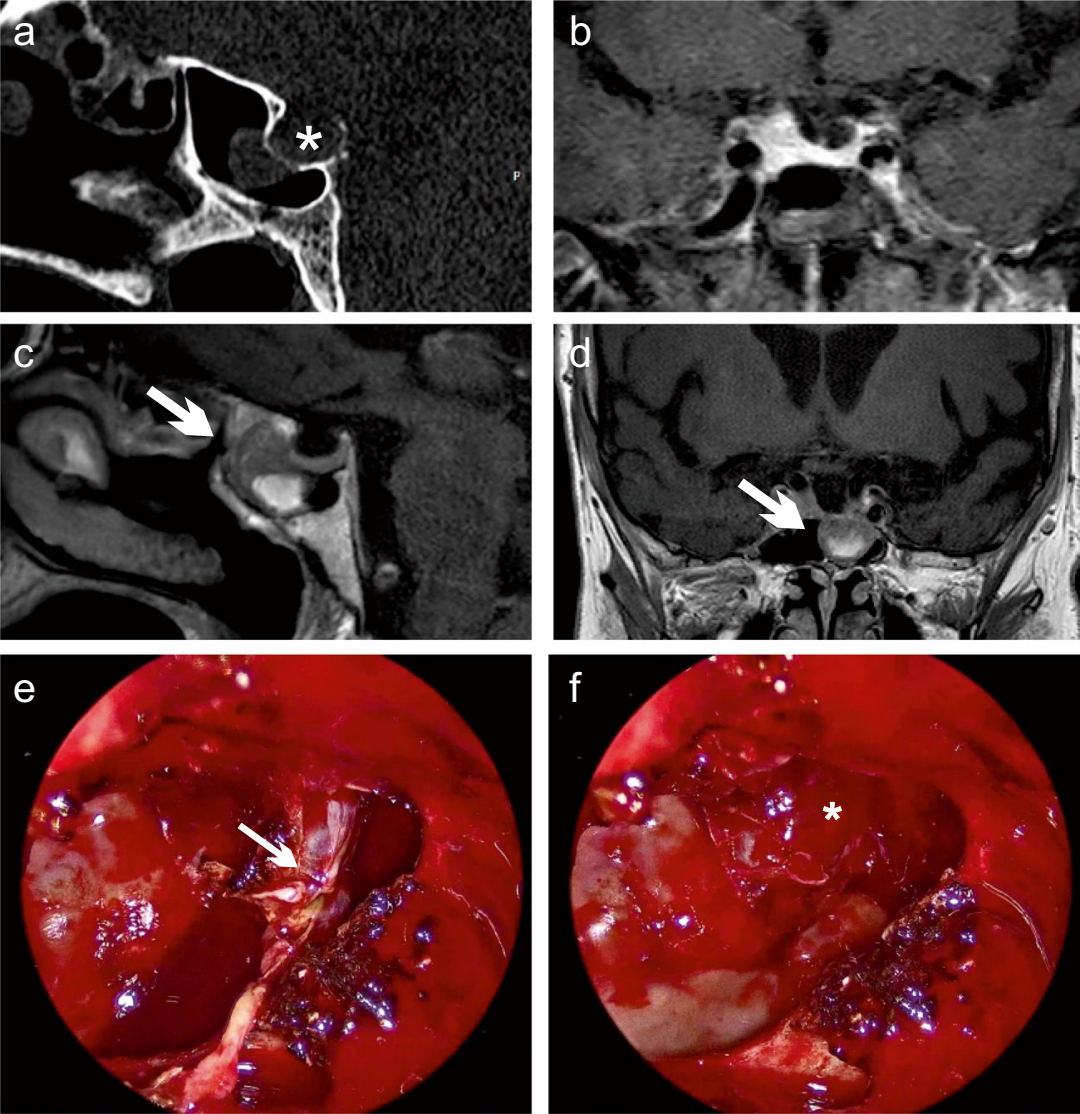
**a** Preoperative sagitally reconstructed CT showing a thinned, but present bony sellar floor (*). **b** Coronal contrast-enhanced MRI images showing a small, narrow pituitary gland. The pituitary stalk can be seen in the middle. **c** Sagittal and **d** coronal contrast-enhanced MRI images showing a mass in the sphenoid sinus (arrow) with heterogeneous contrast enhancement and no evident connection with the sella turcica. **e** Intraoperative images showing a well-circumscribed tumor mass (arrow). **f** Erosion of the sellar wall (*) could be observed after complete tumor resection

An 81-year-old woman was referred to the neurosurgical outpatient clinic with a 1-year history of chronic parietotemporal and frontal headache, predominantly on the left side, that radiated to the neck and shoulder as well as occasional nasal bleeding. No visual impairment or diplopia was reported. The patient had hypertension, obesity (BMI 31 kg/m^2^), and type 2 diabetes. The serum cortisol and plasma ACTH levels were within the low to mid-normal range. Additionally, the patient had a history of hypothyroidism, treated with L-thyroxin, and chronic pain treated with amitriptyline leading to secondary elevated serum levels of prolactin (3007 mU/l; reference range 102–496 mU/l) yet without symptoms of hyperprolactinemia. Computed tomography scan (CT) and magnetic resonance imaging (MRI) showed a space-occupying mass in the sphenoid sinus, with thinning of the sellar floor, but without evident communication to the sellar region (Fig. 1a–d). Intraoperatively, the tumor appeared vascularized and showed sufficient separation from the surrounding tissue (Fig. 1e, f). After complete resection, an osseous erosion of the sellar floor was apparent, yet there was no direct contact between the tumor and the sellar region (Fig. 1f).

**Fig. 2:**
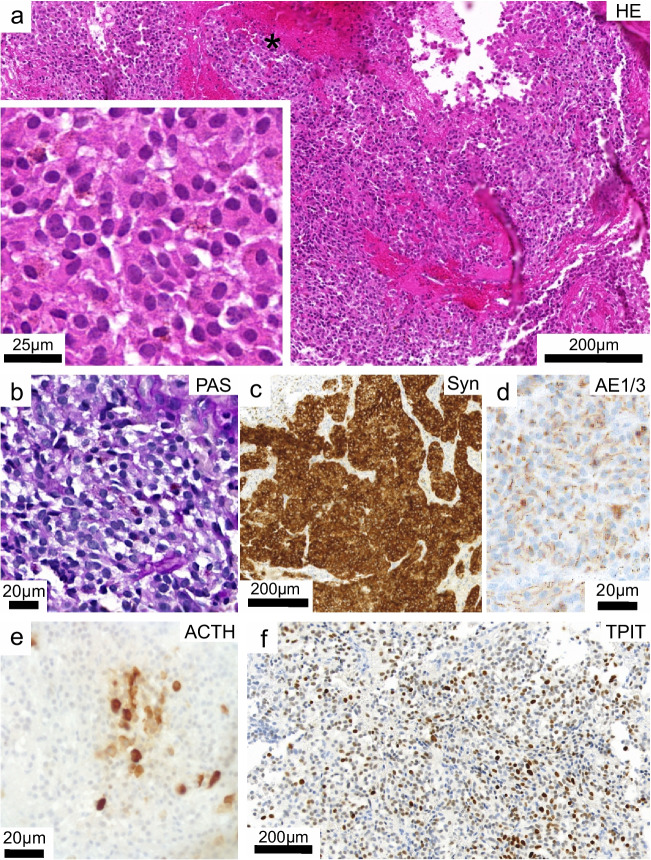
**a** Representative microphotographs of hematoxylin–eosin (H&E) stained tissue sections showing a hypercellular tumor with areas of bleeding (*). At higher magnification (inset), the tumor cells show monomorphic epithelioid morphology and low nuclear atypia. No mitotic figures could be observed. **b** Periodic acid Schiff staining showing a faint cytoplasmic reaction. The tumor cells show a **c** strong expression of synaptophysin and **d** diffuse as well as perinuclear dot-like staining for AE1/3. **e** ACTH was mostly negative with strong expression in scattered cell groups. **f** Immunohistochemistry for the transcription factor TPIT showing multifocal expression with variable intensities

Histologically, we observed a hypercellular tumor with a monomorphic epithelioid morphology with well-defined mostly rounded to polygonal and occasionally elongated cells. The growth pattern was mostly diffuse. The nuclei were predominantly round with minimal chromatin condensation and low nuclear atypia. No mitotic figures could be observed (Fig. 2a). The cytoplasm showed only sparse staining in the periodic acid–Schiff (PAS) reaction (Fig. 2b). The tumor was partially encapsulated with signs of acute and chronic hemorrhage with iron deposition, as well as necrotic areas. Normal-appearing adenohypophysis could not be identified. Immunohistochemically, the tumor cells expressed synaptophysin (Fig. 2c) and showed a perinuclear dot-like expression and focally a diffuse staining with cytokeratin AE1/3 (Fig. 2d). A similar expression was observed in CAM5.2, and there was no evidence of Crooke cells. Overall, the proliferation index was low 2–3% (Mib1) and focally reached up to 5% in hemorrhagic areas, in which an infiltration of macrophages and lymphocytes was observed. Only a few clusters of neoplastic cells expressed ACTH (Fig. 2e). TSH, FSH, LH, prolactin, or hGH were not expressed. The transcription factor for corticotroph differentiation lineage TPIT showed a multifocal expression pattern with a wide range of intensities including a few very intense nuclei up to complete negativity (Fig. 2f). There was no expression of the transcription factors PIT1 and SF1. The histological and immunohistochemical findings prompted the diagnosis of an ectopic sparsely granulated corticotroph PitNET/adenoma. Due to the lack of clinical evidence of cortisol excess, the tumor was moreover termed “silent” ectopic corticotroph PitNET/adenoma.

**Fig. 3:**
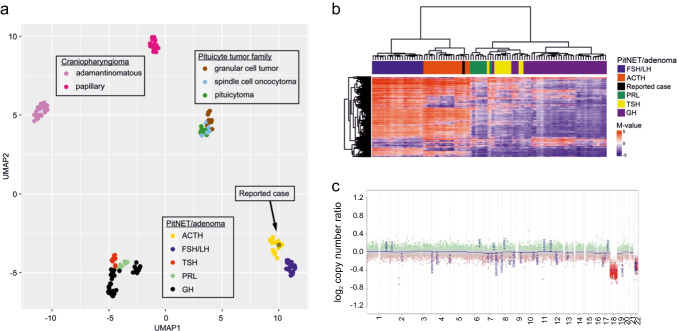
**a** Uniform manifold approximation and projection (UMAP) plot comprising the presented case (⦻) and other tumors of the sellar region [20]. The dimension reduction was performed on the 10,000 most variant CpG sites. The reported case shows an epigenomic profile most similar to sellar corticotroph PitNET/adenoma. **b** Unsupervised clustering confirms a strong epigenomic similarity of our reported case (black) and corticotroph PitNETs of the sellar region, as compared with other PitNET subtypes. Clustering is based on the 1000 most variant CpG sites and Euclidean distance. **c** Copy number variation plots from the reported case showing a loss of chromosomes 18 and 22 in the absence of other evident chromosomal abnormalities. FSH/LH, gonadotroph PitNET/adenoma; ACTH, corticotroph PitNET/adenoma; PRL, lactotroph PitNET/adenoma; TSH, thyrotroph PitNET/adenoma; GH, somatotroph PitNET/adenoma. DNA methylation data was processed using the minfi package in R

We then performed molecular diagnostics using global DNA methylation analysis (EPIC array, 850 k). The methylation profile of the tumor was compared to previously defined methylation classes using the publicly available database of the German Center for Cancer Research (DKFZ) [20] available via www.molecularneuropathology.org. The brain tumor classifier (v12.5) showed an excellent match (score 0.93) with the methylation class “Pituitary Adenoma, Subtype ACTH producing,” suggesting that the epigenomic profile of the tumor matches with sellar corticotroph tumors in spite of its ectopic localization. The similarity of the reported case to sellar corticotroph PitNET was further illustrated by means of a uniform manifold approximation and projection (UMAP) dimension reduction plot and unsupervised cluster analysis (Fig. 3a, b). The copy number variation profile showed loss of chromosomes 18 and 22 without further evident chromosomal abnormalities (Fig. 2c).

The patient was discharged and after 7 month-follow-up no progression of the tumor was seen. The secondary elevated serum levels of prolactin remained after surgery (2681 mU/l; reference range 102–496 mU/l), while the levels of the other pituitary hormones were within a normal range.

## Discussion

In this report, we provide a detailed characterization of an ectopic PiTNET/adenoma and demonstrate the first epigenomic data supporting a common origin of ectopic and sellar PitNET/adenomas. Our findings are limited to a single case report of one ectopic corticotroph lineage tumor.

In our case, the tumor was localized in the sphenoid sinus, one of the most common localizations of ectopic PitNET [2]. The age of presentation in our 81-year-old patient was above the reported mean age of 46–50 years [2, 12, 16, 19].

In the preoperative CT, a thinning of the sellar floor was observed which correlated intraoperatively with bone erosion. Interestingly, the sphenoid bone is frequently subject to erosion not only in sphenoid ectopic PitNET [12, 16, 19, 25] but also in sellar PitNET [12, 16, 19, 25]. Mechanistically, bone erosion depends on the interplay between tumor cells and the appropriate bone microenvironment [26–28], and it has been proposed that osteoclast activation might be induced by a local inflammatory response [27, 29, 30]. In our case, the tumor presented evidence of a chronic reaction with acute and old hemorrhages, necrosis, abundant macrophages, lymphocytic infiltration, and the presence of a fibrous capsule with iron deposition. It is therefore plausible that the local microenvironment represented a favorable substrate for osteoclast activation and resulting bone erosion, as described for sellar PitNET [27, 29].

The expression of transcription factors in ectopic PitNET has not been systematically evaluated thus far. Here, we report the expression of TPIT in a multifocal pattern with a wide range of intensities ranging from a few very intense nuclei up to complete negativity analogous to the pattern described for intrasellar non-secreting PitNET [31]. Also, the expression of cytokeratin AE1/AE3 and Cam5.2 showed a stark similarity to sellar silent TPIT-positive PITNET [32] which was characterized by a perinuclear accumulation and multifocal diffuse expression. These findings suggest that, at least for ectopic silent corticotroph adenoma, transcription factor and cytokeratin expression are similar to silent TPIT-positive PitNET in a sellar location [16, 32].

Moreover, strong similarities could be observed at the epigenetic level. Genome-wide methylation analyses have revealed that epigenomic signatures of PitNET/adenomas closely relate to their pituitary lineages [20, 23, 33], thus reflecting the tumor’s cellular origins and therefore representing a reliable ancillary diagnostic method [20, 34].

The reported ectopic corticotroph PitNET/adenoma displayed a methylation profile most similar to sellar corticotroph PitNET/adenoma, as demonstrated by multiple analytical approaches. Additionally, we could detect a loss of chromosomes 18 and 22, a finding previously reported in sellar PitNET/adenoma [35] and in sellar corticotroph PitNET/adenoma in particular [20]. However, chromosomal aberrations are not specific to PitNET/adenoma types and subtypes [35].

Taken together the immunohistochemical and epigenetic characteristics of the ectopic corticotroph PitNET/adenoma presented in this report point towards a strong biological similarity with sellar corticotroph PitNET/adenoma. Our findings suggest a common developmental origin of ectopic and sellar PitNET/adenomas and spotlight the impact of genome-wide DNA methylation analyses as a powerful tool to aid in the diagnostics of these challenging lesions. Further studies will be needed to evaluate if molecular features of ectopic PitNETs as a group, generally match those of their sellar counterparts.

## Data Availability

Data are available from the corresponding author upon request.
